# Correlates of dental anxiety and phobia in a sample of patients receiving dental care in a dental school setting

**DOI:** 10.21203/rs.3.rs-8058976/v1

**Published:** 2025-12-05

**Authors:** Ifeanyi David Okoye, Eugene Dunne, Sung Woo Lim, Marisol Merchan Tellez

**Affiliations:** 1. Maurice H. Kornberg School of Dentistry, Temple University, Philadelphia, Pennsylvania, USA.

**Keywords:** Dental anxiety, Dental phobia, psychological constructs

## Abstract

**Background:**

Dental anxiety, fear, and phobia are common factors that prevent individuals from seeking dental care by delaying dental care or avoiding the visit entirely, which may cause a decline in oral health-related quality of life. Dental anxiety often has a correlation with other psychological constructs. Our aim was to examine the associations between psychological constructs, dental anxiety, and phobia, and its variations among key demographics among patients seeking dental care in a dental school setting.

**Methods:**

Baseline data from 499 patients who participated in a randomized clinical trial that evaluated the efficacy of an online intervention in managing dental anxiety was used. Subjects completed a semi-structured interview according to the Diagnostic Schedule Manual-IV (DSM-IV) criteria and self-reported measures on dental anxiety (Modified Dental Anxiety Scale), fear or avoidance of dental care (Clinical Severity Rating), sensitivity to pain (Pain Sensitivity Index), ability to tolerate distress (Distress tolerance scale), blood-injection-injury (Fear Questionnaire For Blood Injection Injury), phobia, and other psychological factors. Paired sample t-tests, ANOVA, and Multivariable Regression Models were used for analyses using R 4.3.2. Statistical significance was set at p-value < 0.05.

**Results:**

Mean age of subjects was 48.9±14.7 years old, most were female (71.6%), non-Hispanic (88.6%) with an income lower than $30,000 (40.0%) and most had completed a high school diploma /GED (26%). The majority (63.3%) reported high dental anxiety (MDAS≥19) and 64.57 % met criteria for specific phobia (CSR≥4), with mean scores of 19.53 ± 3.62 and 4.49 ± 1.69 respectively. Mean scores for FQBII, PSI, DTS, and ASI were 15.51 ± 10.15, 68.02±22.9, 44.73±13.2, and 31.0 ± 16.87 respectively. Significant differences in CSR were observed by age (p< 0.01), sex (p= 0.02), and race (p<0.01), while the psychological constructs FQBII, DTS and PSI varied significantly across age, race, and ethnicity (p<0.05).

**Conclusion:**

Dental anxiety scores were higher among participants who were African American, low-income, and women compared to Caucasians. Age, sex, income, and race demonstrated to have a strong association with dental anxiety. Subjects with high pain sensitivity and fear of blood/injections had an increased dental anxiety score.

## INTRODUCTION

Dental anxiety involves an increased level of nervousness and concern about visiting the dentist and receiving dental care (Dou et al., 2018). Dental fear is considered a negative response to perceived threatening stimuli related to dental treatment (Stein et al., 2022), and dental phobia refers to an overwhelming fear of dental visits or procedures, which can significantly impact individuals if they exhibit severe avoidance behavior (Kılıç et al., 2014). This emotional state can have several detrimental effects, such as poor oral health, a predisposition to postpone important dental procedures, and in certain situations, a decline in oral health-related quality of life. Individuals experiencing dental anxiety may find it challenging to maintain regular dental check-ups and may delay essential treatments, ultimately impacting their overall oral health and well-being. It is important to identify and treat dental anxiety to ensure people receive the care they require for a healthy mouth and improved quality of life (Dou et al., 2018).

Dental fear or anxiety is a common factor that makes individuals seeking dental care to delay presentation to the dental clinic or terminate treatment entirely. This fear can make it challenging to proceed with preventive or conservative dental procedures, potentially leading patients to seek emergency care for treatment in the future (Tellez et al., 2015; Tellez et al., 2025). Patients experiencing dental anxiety commonly express negative thoughts and emotions, heightened reliance on medication, disrupted sleep patterns, increased somatic complaints, and diminished social and occupational functioning compared to individuals without dental anxiety (Tellez et al., 2015). In addition, individuals with previous traumatic experiences exhibit severe anxiety. Cognitive behavioral therapy (CBT) has been shown to be effective in reducing avoidance based coping behaviors (Konnerker et al., 2025).

Approximately 10% to 20% of adults in the United States experience dental anxiety. Despite advancements in modern dentistry, the levels of dental anxiety seem to have remained unchanged since the mid-1900s (Tellez et al., 2015). The prevalence of co-morbid phobias such as agoraphobia and social phobias is about 45%, while co-morbid anxiety and mood disorders such as generalized anxiety disorder and social anxiety disorder range from 30% to 43% (Halonen et al., 2012). A noticeable pattern emerges as the degree of dental anxiety rises, so does the probability of experiencing co-occurring phobias and disorders (Halonen et al., 2012).

Hispanic adults had more than double the likelihood of expressing dental anxiety compared to white adults. Similarly, African American, and Asian adults also had higher tendencies to report dental fear in comparison to their white counterparts. In addition, demographic differences play an important role in the prevalence of dental anxiety across adult populations (CareQuest, 2023). Furthermore, about 60% of adults who recorded experiencing dental fear during their most recent dental visit were females, and approximately 3% of adults revealed that their last dental appointment was marked by intense fear and nervousness, which made dental treatment difficult to execute or led to its failure (CareQuest, 2023). Further dental anxiety measurements among various racial populations would aid better epidemiological studies and proper evaluation of dental fear among these populations. However, various anxiety disorders including dental phobia might have cultural variances, so it is critical to understand factors associated with variations in prevalence estimates including cultural aspects. Researchers have pointed out that this would be important as a more significant effort to improve oral health inequalities among various racial groups (Coolidge et al., 2008).

In the context of dental anxiety, several key psychological constructs have been examined, shedding light on the complex interplay between emotional states and oral health. These psychological constructs include oral health-related quality of life, emotion regulation, distress tolerance, mindfulness, and blood-injection-injury (BII) fears. Researchers hypothesized these constructs to be associated with anxiety, phobia severity, and avoidance (Kinner, 2014). Pain (both real and perceived) emerges as a significant factor, with dental anxiety exacerbating the experience of pain and fostering a cycle of avoidance behavior. Distress tolerance has also garnered attention, suggesting that individuals with dental anxiety may possess lower distress tolerance, potentially intensifying their anxiety during dental procedures. Furthermore, the close association between dental anxiety and the fear of blood-injection-injury (BII) is evident, with studies consistently revealing a strong link between the two (Hittner et. al., 2009; Kinner, 2014). This fear extends beyond dental procedures to encompass elements such as needles and injections, contributing to treatment avoidance. Understanding these psychological constructs is paramount for devising effective interventions to alleviate dental anxiety and promote oral health (Kinner, 2014).

Our aim was to examine the associations between psychological constructs, dental anxiety, and phobia, and its variations among key demographics among patients seeking dental care at in a dental school setting.

## MATERIALS AND METHODS

### Enrollment:

This secondary data analysis is a cross-sectional study using baseline data from 499 patients who participated in a randomized clinical trial that evaluated the efficacy of an online intervention in managing dental anxiety (Tellez et al., 2025). The protocol of this study was reviewed and approved by the Institutional Review Board of Temple University. This research was funded by the National Institute of Dental and Craniofacial Research (NIDCR U01DE027328).

Participants enrolled were patients of Temple University Kornberg school of Dentistry (TUKSoD) scheduled for a dental appointment for any non-emergent care and were recruited via the scheduling software and contacted. Patients who indicated interest in the research were contacted by telephone, asked if they were willing to be screened for dental anxiety, and if so, if they would be interested in participating in a dental anxiety study.

### Inclusion and exclusion criteria

To participate in the study, patients had to be between the ages of 18 to 75 years, sufficiently fluent in written and spoken English, be willing to give an informed consent, and have a Modified dental anxiety scale score of ≥ 19 or a score of 4 to 5 on at least 2 of the 5 items. Participants were excluded from the study if they self-report a current medical condition (e.g., cardiopulmonary disease, seizure disorder), if they had current suicidal or homicidal ideation/intent or other condition that would necessitate more clinical attention over an intervention focused on dental anxiety, current psychosis, diminished mental capacity, or other conditions that may significantly diminish the patient’s ability to adequately focus attention adaptively on the current protocol, and inability to provide written informed consent. No restrictions were made based on gender, ethnicity, nor socioeconomic status (Tellez et al., 2025).

### Measures:

Participant information was collected using a semi-structured diagnostic interview conducted by telephone to evaluate the presence and intensity of specific phobias related to dental procedures. MDAS and clinical severity rating (CSR) were the main outcomes. The self-report questionnaire comprised of the following measures.

1. Dental Anxiety: Dental anxiety was assessed using the Modified Dental Anxiety Scale (MDAS), a 5-item scale that evaluates fear related to dental procedures such as cleaning, drilling, and local anesthetic injections. (Humphris et al., 1995; Humphris et al., 2009)
2. Dental phobia, Anxiety and Related Disorders Interview Schedule for DSM-5 (ADIS-5): This semi-structured interview assesses DSM-5 anxiety disorders, including dental phobia. It includes ratings of dental fear, degree of avoidance of dental procedures, and resultant interference were assessed with the aid of Anxiety Disorders Interview Schedule for DSM-5 (ADIS-5). CSR was extracted from ADIS and a score of ≥ 4 suggests that participants meet criteria for a specific phobia (1 to 8). The interview was conducted at baseline assessment and at one- and three-month follow-ups (American psychiatric association, 2013; Brown et al., 2001).
3. Pain: Pain intensity experienced during the last dental visit was measured using the Pain Intensity Numeric Rating Scale (PI-NRS), an 11-point scale ranging from 0 (no pain) to 10 (worst possible pain). Pain Sensitivity Index (PSI) was also measured, and it is a 16-item self-report measure that assesses the fearful appraisal of pain and its expected physical, psychological, and social consequences. It measures pain sensitivity, and it is administered at baseline with the one- and three-month follow-ups. (Gross et al., 1992)
4. Blood-Injection-Injury (BII) Phobia: This was measured using the 5-item subscale of the Fear Questionnaire (FQ-BII). It assesses an individual’s avoidance of situations involving blood, injury, and injections due to anxiety or fear. Cronbach’s alpha for this subscale in the study sample was 0.78 (Marks et al., 1979).
5. Distress Tolerance: Distress tolerance was evaluated using the Distress Tolerance Scale (DTS), a 15-item measure assessing an individual’s perceived ability to experience and tolerate negative emotional states. The scale contains four subscales: tolerance, appraisal, absorption, and regulation, with Cronbach’s alpha of 0.89 in the study sample (Simons et al., 2005).

### Statistical Analysis

Descriptive analyses were first performed to summarize the characteristics of the study population. Following this, univariate and bivariate analyses were done to explore both the characteristics of individual variables and correlations between two study outcomes (MDAS, CSR) and key psychological constructs, and demographic factors (using paired sample t-test and ANOVA). A sub-group comparison of MDAS and CSR by demographics using Tukey HSD test was also conducted. Subsequently, multivariable linear regression was used to assess associations between dental anxiety/dental phobia and selected demographics and psychological constructs, while controlling for other variables in the model. These analyses were performed using R 4.3.2. The predetermined threshold for statistical significance was set at two-sided p-values < 0.05.

## RESULTS

### Demographic Characteristics:

A total of 499 subjects were recruited for the study and demographic data were collected. Table 1 shows the demographic characteristics of the participants. The age range of the participants were 18 to 75 years (48.9±14.9), with the majority identifying as female (71.7%) and African American (62.45%). Most were middle-aged (43.3%), employed (39%), completed high school (26%), were low-income earners with an annual income below $30,000 (40%), and were single (55%) in terms of marital status. In addition, 11.4% of the total sample were Hispanic/Latinos.

As seen in table 2, the mean Modified Dental Anxiety Scale (MDAS) was 19.53 ± 3.62, with a range 8 to 25. A score of ≥ 19 = high anxiety, and 63.30% of participants were highly anxious. The mean CSR was 4.49 ± 1.69, with a range of 1 to 8. Almost 65% of the subject met criteria for dental phobia. Moreover 50.0 % of participants were highly sensitive to anxiety based on the ASI.

### Bivariate Analysis

Study outcomes (MDAS, CSR), and psychological constructs (FQBII, DTS, PSI, ASI)) were stratified by selected demographic variables (Tables 3 and 4).

There were statistically significant differences in the mean CSR scores across the different age groups (*p*< 0.01), with the middle-aged individual showing a CSR mean score of 4.73 ±1.65.

There were statistically significant differences in the mean MDAS and CSR scores by gender (p< 0.01 and 0.03 respectively) and race. For example, white or Caucasian individuals had a higher CSR mean score of 4.73 ± 1.65 compared to the other races (*p*=0.003). Income showed statistically significant differences in the MDAS with a *p =* 0.04.

There were statistically significant differences in the mean score of FQBII, with young adults showing the highest mean score of 18.36 ± 9.83 (*p*< 0.01), in DTS, as older adults had the highest mean (*p*< 0.01). Furthermore, young adults showed the highest PSI score (p< 0.01).

Considering race, there were statistically significant differences in the mean FQBII, with Asians showing a higher mean FQBII of 21.73 ± 10.05 compared to other racial groups (*p* < 0.01). Black or African Americans showed the highest DTS mean of 46.44 ± 12.66 (*p* < 0.01). Furthermore, there was statistically significant difference in the PSI, with the Asians showing the highest PSI of 80.05 ± 18.61 (*p* < 0.01). Finally, there were no statistically significant differences in mean FQBII, DTS, PSI, and ASI by income. However, Hispanics had a higher mean FQBII than non-Hispanics (*p* = 0.01). Mean DTS was statistically significant different by ethnicity, with the non-Hispanics showing the highest mean 45.58 ± 2.97 (*p* < 0.01). In addition, PSI varied by ethnicity. For example, Hispanics had the highest PSI and ASI mean scores (*p*< 0.01).

### Multivariable Analysis

We found that black individuals had a 0.64 increase in MDAS when compared to Whites after adjusting for other demographic and clinical variables in the model (Table 5).

For every one unit increase in FQBII, there was a 0.06 increase in MDAS after adjusting for other variables in the model (p<0.01). In addition, PSI was also found to be significantly correlated to dental anxiety. For every one unit increase in PSI, there was a 0.06 increase in MDAS after adjusting for other variables in the model (p<0.01). In contrast, both DTS and ASI seemed to decrease MDAS scores but did not reach statistical significance. The model explained 22% variability in MDAS scores.

We also found that both young and middle-aged adults had a 0.45 and 0.71 increase in mean CSR when compared to the older adults after adjusting for other key demographics in the model (*p*-value = 0.04 and < 0.01 resp.).

Furthermore, Black participants had a 0.54 decrease in mean CSR when compared to Whites after adjusting for other key demographics in the model (*p*-value <0.01). In addition, individuals with income ≥$100,000 showed a 0.67 decrease in mean CSR compared to individuals with an income <30,000 after adjusting for other key demographics in the model (*p*-value = 0.03). However, sex was not significantly associated with CSR. The model only explained 6 % of the variability in CSR scores.

## DISCUSSION

Dental anxiety and dental phobia are common conditions that can lead to refraining from dental treatment (Tellez et. al., 2015; Locker, D. et al., 2003) and overall can lead to a decrease in oral health and oral health quality of life. These conditions often result in the need for more complex and costly dental care. Additionally, they are associated with various psychological symptoms (Kinner, 2014) such as heart palpitations, dry mouth, sweatiness, syncope, signs of panic.

About 10 to 20% of American adults experience dental anxiety and fear (Tellez et al., 2025). and despite advances in the field of dentistry, there has been limited progress in implementing effective, non-pharmacological intervention in dental settings. In addition, psychological interventions based on cognitive behavioral therapy have shown to be highly effective in the improvements of dental anxiety in dental settings (Tellez et al., 2025). Researchers have hypothesized that some psychological constructs such as blood-injection-injury (BII) fear, distress tolerance, pain sensitivity, anxiety sensitivity, social appearance, oral health-related quality of life, emotional regulation, and mindfulness symptoms are linked to anxiety severity, phobia levels, and avoidance behaviors (Locker et. al., 2003; Mehrstedt et. al., 2007; Kinner, 2014). We have examined four key psychological constructs which are blood-injection-injury (BII) fears, distress tolerance, pain sensitivity, anxiety sensitivity in relation to dental anxiety (MDAS), and anxiety-related disorder (ADIS).

In the current study, we hypothesized that there are correlations between dental anxiety and psychological constructs and correlations between dental anxiety and demographic factors.

The results of this secondary analysis supported our hypothesis, such that dental anxiety was related to several demographic and psychological constructs. Dental anxiety among age groups revealed that middle-aged individuals were more anxious compared to other age groups; however, this was not statistically significant (*p*= 0.064). This finding was similar to studies by Alansaari et al, 2023, Stabholz et al, 1999, Thomson et al. 1996, and Vassend, 1993. However, Malvania et al., in 2011 found that young adults showed higher levels of dental anxiety compared to other groups. Furthermore, the middle-aged adults demonstrated a significantly higher level of dental phobia (CSR) than the other age groups (*p*< 0.01). This suggests that middle-aged individuals have a higher level of avoidance compared to the other age groups. Moreover, Doerr et al (1998) found no correlation between age and dental anxiety. Females exhibited higher levels of dental anxiety and dental phobia compared to males. This finding was consistent with prior studies (Malvania et al., 2011; Alansaari et al., 2023; Doer et al. 1998), but it was not statistically significant in their studies.

Considering race, Black participants exhibited higher levels of dental anxiety compared to other races. This was in keeping with findings by (Alansaari et al., 2023); however, Doerr et al in 1998 found no correlation between race and dental anxiety. Regarding income, individuals who earned $30,000 - $59,999 showed the highest level of dental anxiety compared to other income groups. However, Alansaari et al. in 2023 did not find any significant differences by income.

Significant demographics that correlate to dental anxiety (sex and income), and CSR (age and race) were consistent with a previous study (Alansaari et al., 2023).

In addition, age, race, fear of blood-injection-injury, and sensitivity to pain were found to be significantly correlated to dental anxiety. The findings of this study were similar to those of previous studies (Tellez et. al., 2015). However, the amount of variability unexplained by the models is still substantial. Furthermore, we found a positive correlation between dental anxiety and fear of blood-injection-injury/sensitivity to pain. This was similar to findings suggested by previous studies (Vika et al., 2008; Vassend, 1993).

In the present study, anxiety sensitivity was not significantly correlated to dental anxiety nor phobia, this is consistent with findings previous studies (De Jongh et al., 1995).

Studies have revealed that different assessments of dental anxiety and specific phobia of dental procedures may lead to inconsistent findings regarding the relationship between dental anxiety and other psychological disorders. Inconsistent results could be due to varying diagnostic instruments used, such as self-report measures versus clinician-administered interviews, and differences in sample populations, such as individuals seeking treatment at dental clinics versus community samples. Most of the studies investigating the correlations of dental anxiety have employed self-report measures, rather than clinician-administered interviews, which may not accurately assess specific phobia of dental procedures across the literature. However, we employed the use of both self-reported measures and clinical interviews to acquire more accurate baseline information and the relationship between dental anxiety and psychological constructs. The cross-sectional nature of pain data should be interpreted cautiously, as more anxious patients could perceive aversive situations as more traumatic than non-anxious patients.

Further research is needed to refine our comprehension of these relationships and to develop tailored strategies for managing distress, pain sensitivity, and blood-injection-injury related fears in individuals with dental anxiety. To prioritize the development of targeted interventions to improve dental anxiety and phobia, especially among non-white, low-income women, while also assessing the effectiveness of culturally sensitive approaches.

As our study limitation, it is important to highlight that the individuals recruited for this study were patients actively seeking care in the dental school, and their responses may differ from those in the general population. This might consequently limit the generalization of the research findings.

## CONCLUSION

This study showed that dental anxiety and phobia were significantly associated with key demographics and psychological constructs. Dental anxiety and phobia were highly prevalent in this clinic sample, being more prevalent among non-white low-income women. Age, sex, income, and race were found to have a positive correlation with dental anxiety. Furthermore, psychological constructs such as Blood-Injection-Injury and pain sensitivity index were also found to be predictors of dental anxiety. However, having high pain sensitivity and fear of blood/injections seemed to increase dental anxiety.
